# Mediterranean diet score and total and cardiovascular mortality in Eastern Europe: the HAPIEE study

**DOI:** 10.1007/s00394-015-1092-x

**Published:** 2015-11-17

**Authors:** Denes Stefler, Sofia Malyutina, Ruzena Kubinova, Andrzej Pajak, Anne Peasey, Hynek Pikhart, Eric J. Brunner, Martin Bobak

**Affiliations:** 1 Department of Epidemiology and Public Health, University College London, 1-19 Torrington Place, WC1E 6BT London, UK; 2 Institute of Internal and Preventive Medicine, Siberian Branch of the Russian Academy of Medical Sciences, Novosibirsk State Medical University, B. Bogatkova 175/1, Novosibirsk, Russia 630089; 3 National Institute of Public Health, Šrobárova 48, 100 42 Prague, Czech Republic; 4 Department of Epidemiology and Population Sciences, Jagiellonian University, Grzegorzcka 20, 31-531 Kraków, Poland

**Keywords:** Diet, Nutrition, Mortality, Prevention, Eastern Europe

## Abstract

**Purpose:**

Mediterranean-type dietary pattern has been associated with lower risk of cardiovascular (CVD) and other chronic diseases, primarily in Southern European populations. We examined whether Mediterranean diet score (MDS) is associated with total, CVD, coronary heart disease (CHD) and stroke mortality in a prospective cohort study in three Eastern European populations.

**Methods:**

A total of 19,333 male and female participants of the Health Alcohol and Psychosocial factors in Eastern Europe (HAPIEE) study in the Czech Republic, Poland and the Russian Federation were included in the analysis. Diet was assessed by food frequency questionnaire, and MDS was derived from consumption of nine groups of food using absolute cut-offs. Mortality was ascertained by linkage with death registers.

**Results:**

Over the median follow-up time of 7 years, 1314 participants died. The proportion of participants with high adherence to Mediterranean diet was low (25 %). One standard deviation (SD) increase in the MDS (equivalent to 2.2 point increase in the score) was found to be inversely associated with death from all causes (HR, 95 % CI 0.93, 0.88–0.98) and CVD (0.90, 0.81–0.99) even after multivariable adjustment. Inverse but statistically not significant link was found for CHD (0.90, 0.78–1.03) and stroke (0.87, 0.71–1.07). The MDS effects were similar in each country cohort.

**Conclusion:**

Higher adherence to the Mediterranean diet was associated with reduced risk of total and CVD deaths in these large Eastern European urban populations. The application of MDS with absolute cut-offs appears suitable for non-Mediterranean populations.

**Electronic supplementary material:**

The online version of this article (doi:10.1007/s00394-015-1092-x) contains supplementary material, which is available to authorized users.

## Introduction

Mediterranean diet is the traditional eating pattern of populations around the Mediterranean Sea in Southern Europe [1]. It is usually characterized by high consumption of fruits, vegetables, legumes, cereals, fish and olive oil, low consumption of milk and meat and moderate intake of alcohol [2, 3]. Mediterranean diet has found to be protective against cardiovascular disease (CVD) and other chronic conditions in numerous observational epidemiological studies, as well as in primary and secondary prevention trials [4–6]. Mediterranean diet score (MDS), the indicator of adherence to the Mediterranean diet, based on consumption of selected foods, was first introduced by Trichopoulou in 1995 [7], and several modified versions of the original score have been developed since then [3].

The associations between MDS and mortality outcomes have been investigated primarily in Southern European countries and less frequently in non-Mediterranean populations. One small study (*n* = 411) in Eastern Europe among elderly people was inconclusive [8].

An important disadvantage of MDS definitions used previously is that component scores for diet composition are based on sample-specific cut-off values (usually sex-specific medians). Scoring can differ substantially between study samples and may not reflect meaningful differences between healthy and unhealthy food intakes [9]. In addition, relative cut-off points do not allow comparison of MDSs between populations. For these reasons, Sofi et al. developed a scoring system which uses absolute cut-off values determined by a review of food intake distributions in previous MDS studies [10]. This new MDS has the potential to overcome the limitations of previous versions of MDS, but it has not been tested in relation to disease outcomes.

The aim of our study was to assess the association between the Mediterranean diet and total, CVD, coronary heart disease (CHD) and stroke mortality in large Eastern European populations using the MDS with absolute cut-off values as proposed [10].

## Methods

### Study design and subjects

We conducted a prospective cohort study based on the Health Alcohol and Psychosocial Factors in Eastern Europe (HAPIEE) project. The details, including the methods applied for dietary and mortality data collection, have been described previously [11–13].

Briefly, the baseline data collection took place between 2002 and 2005 in Krakow (Poland), Novosibirsk (Russia) and six cities in the Czech Republic. Overall, 28,945 middle-aged man and women were randomly selected from population/electoral registers (response rate 59 %). Participants completed a comprehensive questionnaire, provided blood sample and underwent a brief medical examination. All participants signed informed consent. The study protocols were approved by ethical committees at University College London and at each participating centre.

### Dietary assessment

Dietary data were collected with semi-quantitative food frequency questionnaires (FFQs), covering 136, 148 and 147 food and drink items in the Czech, Polish and Russian cohorts, respectively. Using a nine-point scale, participants indicated how frequently a particular food or drink item was consumed over the previous 3 months. Daily intakes of the different food groups were calculated using the EFSA food classification system [14] and country-specific portion sizes, and the McCance and Widdowson Food Composition Database was used to calculate nutrient intake levels [15]. The relative validity of the FFQ data regarding fruit, vegetable and micronutrient intakes was assessed by estimating correlations with concentration biomarkers in a random sub-sample of study subjects. Pearson’s partial correlation coefficients between fruit intake and vitamin C and beta-carotene plasma concentrations in the pooled HAPIEE sample were 0.29 and 0.05, respectively. The correlation coefficients for vegetable intake were 0.11 and 0.17, respectively [13].

The MDS applied in this study followed the recommendations of Sofi et al [10] who defined absolute cut-off values for all MDS components and applied a three-tier scoring system with zero, one or two points given to participants for each component (Table 1). The component regarding olive oil usage had to be modified because the corresponding question in the FFQ did not allow distinction between occasional, frequent and regular users. We gave one point for this component to those participants who stated that they used olive oil for cooking, and zero point to those who reported to cook with any other type of oil. As a result, after adding up the individual component scores, overall MDS ranged from zero to 17.

**Table 1:** Scoring criteria of the MDS components and the percentage of participants with maximum MDS component scores

Components	Scoring criteria of MDS components			% of participants with maximum^a^ component scores							
				Czech		Polish		Russian		Total	
	0 point	1 point	2 points	Males (*n* = 2648)	Females (*n* = 3319)	Males (*n* = 3083)	Females (*n* = 3460)	Males (*n* = 3056)	Females (*n* = 3767)	Males (*n* = 8787)	Females (*n* = 10,546)
Vegetables (g/day)	<100	100–250	>250	21.3	35.9	29.5	32.8	39.0	41.4	30.4	36.9
Fruits and nuts (g/day)	<150	150–300	>300	37.9	59.1	33.0	45.5	8.2	15.0	25.9	38.9
Legumes (g/week)	<70	70–140	>140	60.1	58.4	42.9	38.0	29.6	29.6	29.7	27.1
Cereals (g/day)	<130	130–195	>195	67.9	59.1	80.3	75.7	87.9	73.2	79.2	69.6
Fish (g/week)	<100	100–250	>250	34.1	31.1	42.6	33.0	36.9	33.1	38.0	32.4
Meat and meat products (g/day)	>120	80–120	<80	15.1	30.1	9.5	22.6	9.7	21.0	11.3	24.4
Dairy products (g/day)	>270	180–270	<180	55.9	40.3	46.8	34.3	52.7	49.0	51.6	41.4
Alcohol (g/day)	>24	<12	12–24	16.7	5.9	10.4	1.5	19.9	2.0	15.6	3.0
Olive oil usage	Not used for cooking	Used for cooking	–	5.6	6.0	41.7	40.0	0.3	0.3	16.4	15.1

^a^One point for olive oil usage and two points for all other components

### Mortality follow-up

Linkage with regional or national death registers was used to identify mortality in the sample. Deaths from CVD (ICD-9: 390–459; ICD-10: I00–I99), CHD (410–414; I20–I25) and stroke (430–438; I60–I69) were determined using the 9th and 10th revision of the International Classification of Diseases [16]. There were 70 individuals who died over the course of follow-up with unknown cause of death. These participants were included in the analysis if the outcome was total mortality, but excluded when the associations with CVD, CHD and stroke mortality outcomes were assessed.

### Analytical sample

In order to avoid reverse causation, we excluded all subjects with prevalent CVD or diabetes at study baseline (*n* = 6525). Participants with missing follow-up data (*n* = 1048), missing FFQ data for more than 10 % of the listed items (*n* = 685), extreme energy intake reporters (basal metabolic rate vs. reported energy intake ratio in the top and bottom 1 % of the distribution) (*n* = 548) and those who stated that the FFQ was not representative to their diet (*n* = 806) were also omitted from the analysis. After these procedures, we included 19,333 participants in the study.

### Multiple imputation of missing covariate data

A total of 3106 individuals (16 % of the analytical sample) had missing data on at least one of the following variables: marital status, smoking habits, alcohol intake, education, household amenities score, physical activity, BMI, vitamin supplement intake, mean arterial blood pressure, serum cholesterol level or olive oil usage. Missing at random assumption was considered reasonable because sensitivity analysis showed that “missingness” was significantly associated with several covariates and the results of the analysis on non-imputed data set, using listwise deletion technique, did not differ considerably from our main findings. Ten imputed data sets were created using the “mi impute chained” command in STATA v.13.1 [17, 18]. Age, sex, country cohort, follow-up time and all-cause mortality were applied as predictor variables.

### Statistical analysis

Participants’ adherence to the Mediterranean diet was classified as low (0–7 points), moderate (8–10 points) and high (11–17 points) according to their MDS. These categories reflect similar fraction of the maximum score as those applied by Trichopoulou et al. [2] in the most commonly used scoring system with the maximum of nine points.

The associations between the MDS and mortality outcomes were assessed using Cox proportional hazard models with MDS as both a categorical and a continuous variable. In the latter case, the associations of mortality risk with 1 SD increase in the MDS were calculated. One SD in the MDS was equal to 2.2 points in the pooled sample. Proportionality assumptions were tested with Schoenfeld residuals. The proportion of deaths which could be prevented if participants in the lowest two MDS categories increased their adherence to the Mediterranean diet one category upwards was calculated using a formula applied in previous studies [19] and modified for three exposure categories. Since the dietary assessment methods in the three cohorts were very similar and there was no interaction between MDS and cohort, sex or smoking status, the associations of MDS with mortality were estimated in the pooled sample (but cohort-specific results are presented in supplementary material).

In multivariable models, the associations were adjusted for age, sex, cohort, education (primary or less, vocational, secondary, university), household amenities score (number of household amenities possessed; 0–5: low, 5–7: moderate, 8–12: high), marital status (married/cohabiting, single/divorced/widowed), smoking (non-, ex-, current smokers), physical activity (inactive, moderately active, active; based on cross-tabulating the sex-specific quartiles of leisure time physical activity expressed in MET hours/day with occupational activity categories) [20], total energy intake (MJ/day) and vitamin supplement intake (no intake, irregular intake—<3 times a week, regular intake—at least 3 times a week).

In order to assess the impact of the individual components to the overall MDS, the associations between the MDS component scores and mortality outcomes were also calculated. Multivariable-adjusted HR per one-point increase in each component scores is presented.

All statistical analyses were carried out using the 13.1 version of the statistical software STATA (StataCorp, Texas, USA).

## Results

### MDS components

The proportions of participants in the three cohorts who scored the maximum points for the various MDS components are shown in Table 1. While high proportion of participants scored maximum points for cereal intake in all three country cohorts, less than 25 % of all subject reached this “ideal intake” category regarding meat and alcohol intake and olive oil usage. Adequate intake of fruits and nuts and olive oil was especially rare among Russians. Although the proportion of participants with adequate vegetable, fruit and nut and meat consumption was higher in females than males, for all other MDS components, maximum score was more common in males.

### MDS categories and bivariate results

Table 2 shows the distribution of the sample characteristics across the three MDS categories. Overall, 25 % of the participants had high (>10) MDS. The proportion of these high scorers was the largest in the Polish cohort and smallest among Russians.

**Table 2:** Characteristics of the study sample by MDS categories

	MDS categories			*p* value (trend)^b^
	Low (0–7 points)	Moderate (8–10 points)	High (11–17 points)	
Number of participants^a^	4790	8941	4589	
Cohorts				
Czech (%)	26.1	28.0	36.8	<0.001
Polish (%)	23.9	33.9	41.8	<0.001
Russian (%)	50.0	38.1	21.5	<0.001
Socio-demographic characteristics				
Mean age (SD), years	56.9 (7.1)	57.1 (7.0)	56.7 (6.9)	0.216
Sex: females (%)	49.8	55.8	58.5	<0.001
Marital status: married (%)	73.8	75.4	77.1	<0.001
Education: primary or less (%)	10.3	10.1	9.1	0.045
Education: university (%)	25.8	25.5	27.5	0.068
Household amenities score: low (%)	24.3	21.7	17.1	<0.001
Household amenities score: high (%)	29.2	33.0	38.5	<0.001
Lifestyle characteristics				
Mean energy intake (SD), MJ/day	9.3 (3.1)	9.7 (3.1)	10.0 (3.1)	<0.001
Smoking: current smokers (%)	33.7	30.0	27.2	<0.001
Physical activity: low (%)	49.8	48.8	48.4	0.188
Vitamin suppl. intake: regular (%)	13.9	17.5	22.7	<0.001
Mean healthy diet indicator (SD)	50.9 (8.4)	54.9 (8.6)	57.4 (8.8)	<0.001
CVD risk factors				
Mean BMI (SD), kg/m^2^	27.8 (4.8)	27.8 (4.7)	27.7 (4.6)	0.260
BMI > 30 kg/m^2^ (%)	28.9	28.2	27.1	0.042
Mean arterial pressure (SD), mmHg	105.3 (15.6)	104.8 (15.0)	104.2 (15.5)	0.001
Hypertension^c^ (%)	46.6	47.1	45.1	0.148
Mean total cholesterol (SD), mmol/l	6.0 (1.2)	6.0 (1.2)	5.9 (1.2)	<0.001
Hypercholesterolemia^d^ (%)	76.5	76.8	75.9	0.507
Mortality outcomes				
All-cause, per 1000 person-years	12.2	9.0	7.3	<0.001
CVD, per 1000 person-years	4.3	3.3	1.9	<0.001
CHD, per 1000 person-years	2.4	1.7	0.9	<0.001
Stroke, per 1000 person-years	1.2	0.8	0.4	<0.001

^a^ Including participants with complete MDS data only

^b^ *p* values were calculated by logistic or linear regression, applying covariates as outcome and MDS categories as continuously treated exposure variables

^c^ Mean arterial pressure >110 mmHg or on antihypertensive medication

^d^ Serum cholesterol level >5.2 mmol/l or on lipid-lowering medication

Female sex, married life, high household amenities score, high total energy and regular vitamin supplement intake were related to high MDS. The proportion of smokers was lower among those with high MDS, and not surprisingly, mean healthy diet indicator score (primarily a nutrient-based diet quality index) [13] increased sharply with increasing MDS. Mean arterial blood pressure and total cholesterol level were the lowest in the highest MDS category, and we found clear inverse trend of crude total and cause-specific mortality rates across MDS categories.

### Cox regression results

The simple and multivariable-adjusted associations of MDS with total and cause-specific mortality outcomes in the pooled sample are shown in Table 3. In the multivariable-adjusted models, 1 SD (which equals to 2.2 points) increase in the MDS was significantly associated with reduced risk of total and CVD deaths even if all possible confounders were taken into account. The association with CHD and stroke mortality was also inverse but statistically non-significant. The preventable proportion of deaths was the highest for stroke mortality.

**Table 3:** Results of Cox regression analysis between MDS and mortality outcomes on the pooled sample

Cause of death	dead/*n*	Model	MDS categories^a^						PP (%)^b^	Per 1SD^c^ increase in MDS score		
			Low	Moderate		High		Trend *p* value				
			HR	HR	(95 % CI)	HR	(95 % CI)			HR	(95 % CI)	*p* value
Any-cause	1314/19,333	model1	1.0	0.79	(0.70–0.90)	0.72	(0.62–0.85)	<0.001	11.2	0.87	(0.82–0.92)	<0.001
		model2	1.0	0.85	(0.75–0.96)	0.85	(0.73–1.00)	0.027	5.6	0.93	(0.88–0.98)	0.012
CVD	438/19,263	model1	1.0	0.87	(0.70–1.07)	0.64	(0.48–0.86)	0.003	14.3	0.83	(0.75–0.92)	<0.001
		model2	1.0	0.94	(0.76–1.16)	0.78	(0.58–1.05)	0.118	8.1	0.90	(0.81–0.99)	0.036
CHD	226/19,263	model1	1.0	0.88	(0.66–1.17)	0.64	(0.42–0.96)	0.038	14.3	0.82	(0.71–0.94)	0.004
		model2	1.0	0.97	(0.72–1.29)	0.81	(0.53–1.23)	0.376	6.8	0.90	(0.78–1.03)	0.132
Stroke	109/19,263	model1	1.0	0.86	(0.57–1.30)	0.64	(0.35–1.16)	0.146	14.4	0.84	(0.69–1.03)	0.093
		model2	1.0	0.88	(0.58–1.34)	0.71	(0.39–1.30)	0.273	11.2	0.87	(0.71–1.07)	0.201

Model 1: adjusted for sex, age and cohort

Model 2: adjusted for sex, age, cohort, smoking, education, household amenities score, marital status, energy intake, physical activity and vitamin supplement intake

^a^As in Table 2

^b^Preventable proportion of death if participants in the lowest two categories increased their adherence to Mediterranean diet one category upward

^c^1SD = 2.2 MDS points

Country-specific analyses revealed inverse but not statistically significant associations between MDS and most mortality outcomes in individual cohorts (Table S1 in Online Resource). When the age- and sex-adjusted mortality differences between the Polish and Russian cohorts (the two cohorts with the highest and lowest adherence to the Mediterranean diet) were further adjusted for the MDS, HRs for total and CVD mortality rates reduced by 17.9 and 14.5 %, respectively (data not shown).

We also examined the relationship between the original MDS based on sex-specific median cut-offs for component scores [2] and mortality outcomes (Table S2 in Online Resource). The results suggested somewhat weaker associations. The agreement between the two scores was moderate: Spearman’s correlation coefficient was 0.69, and the linear weighted kappa between the three MDS categories in each score was 0.50.

When the MDS components were examined separately, mortality risks showed inverse tendencies with all components except for meat and olive oil (Table 4). However, most associations were not significant, which suggests that the MDS is a better predictor of mortality than the individual components.

**Table 4:** Results of the Cox regression analysis for the association between MDS component scores and mortality outcomes

Components	Mortality outcomes							
	All-cause		CVD		CHD		Stroke	
	HR^a^	(95 % CI)	HR^a^	(95 % CI)	HR^a^	95 % CI	HR^a^	95 % CI
Vegetables	0.95	(0.87–1.04)	0.88	(0.76–1.03)	0.90	(0.73–1.11)	0.75	(0.55–1.03)
Fruits and nuts	0.97	(0.90–1.05)	0.82	(0.71–0.95)	0.87	(0.70–1.07)	0.68	(0.50–0.94)
Legumes	0.95	(0.89–1.01)	0.96	(0.86–1.08)	1.04	(0.89–1.22)	0.84	(0.66–1.05)
Cereals	0.91	(0.82–1.00)	0.94	(0.79–1.12)	0.90	(0.70–1.16)	1.13	(0.76–1.66)
Fish	0.95	(0.88–1.03)	0.96	(0.83–1.10)	0.87	(0.72–1.05)	1.12	(0.85–1.49)
Meat and meat products	1.00	(0.93–1.09)	1.07	(0.93–1.22)	1.02	(0.84–1.23)	1.25	(0.95–1.62)
Dairy products	0.98	(0.92–1.05)	0.97	(0.86–1.09)	0.98	(0.83–1.16)	0.92	(0.73–1.16)
Alcohol	0.90	(0.80–1.00)	0.89	(0.74–1.07)	0.82	(0.64–1.05)	0.86	(0.58–1.28)
Olive oil usage	1.11	(0.92–1.34)	1.07	(0.73–1.55)	1.23	(0.71–2.16)	1.58	(0.72–3.50)

^a^Per one-point increase in the component score; all HRs are adjusted for age, sex, cohort, education, marital status, household amenities score, smoking, physical activity, total energy intake and vitamin supplement intake

## Discussion

### Main findings

This study in three large urban population samples in Central and Eastern Europe and the former Soviet Union found statistically significant inverse associations between Mediterranean diet score and total and CVD mortality. Using a MDS with absolute cut-off values for component scores, we also found that high adherence to Mediterranean diet, an indicator of healthy eating habits, in this Eastern European sample was rare.

### Limitations and strengths of the study

Several limitations need to be taken into account when the results are interpreted. First, the moderate response rates and the fact that cohorts were restricted to urban settings limit the generalizability of our findings and introduce selection bias. Although mortality rates in the three country cohorts were similar to national-level data [21], dietary habits of the study samples might differ from the Czech, Polish and Russian general populations. For example, previous study suggests that inadequate fruit and vegetable intake is probably more common in rural FSU populations than in those who live in cities [22]. The lack of national representativeness, however, does not affect the internal validity of the findings regarding the association between MDS and mortality.

The second limitation of the study is related to measurement error of dietary intakes and the disadvantages of FFQ [23, 24]. It is likely that the consumption of some foods was over- (i.e. fruits and nuts, vegetables) or underestimated (i.e. alcohol); consequently, the estimated MDS of participants may be imprecise. However, this issue is common to the majority of nutritional epidemiological studies which use an FFQ for dietary assessment. The relative validity of FFQ data in HAPIEE study regarding the intakes of fruits, vegetables and selected micronutrients has been assessed previously using plasma biomarkers, indicating satisfactory correlations [13].

Finally, due to differences in the composition of food groups, food preparation techniques and meal patterns, MDS might have important limitations in measuring the adherence to the exact Mediterranean-style diet in non-Mediterranean population samples [25]. These issues will need to be taken into account in future attempts to assess more precisely the extent to which the Mediterranean-style diet is followed by non-Mediterranean populations.

On the other hand, the prospective study design and the large sample size are important strengths of this analysis. No previous study on this scale has examined the association between MDS and mortality in Central and Eastern Europe or the Former Soviet Union, and this is the first study that applied the MDS with absolute cut-offs, developed by Sofi et al. [10], for such assessment in any population.

### Results in context

The findings of this study are consistent with previous evidence which suggests that Mediterranean diet reduces the risk of CVD and total mortality [2, 5, 26]. Our results also indicate that the literature-based MDS developed by Sofi and colleagues is a good indicator of healthy diet and predicts mortality outcomes well. In a previous analysis, we found that the healthy diet indicator (HDI), a diet quality score that measures the adherence to the World Health Organization’s dietary guidelines, also performed reasonably well in predicting CVD and CHD mortality in the same HAPIEE cohorts [13]. While both analyses suggest that unhealthy diet is an important risk factor for CVD mortality in Eastern European populations, in contrast to HDI, MDS is a food-based score which makes the current results easier to translate into public health recommendations. Previous analysis of the Polish arm of the HAPIEE study also found lower risk of metabolic syndrome in individuals with high adherence to the Mediterranean diet [27], which suggests that the protective effect is at least partly mediated by the favourable metabolic profile.

The MDS with absolute cut-offs appears to be suitable to assess the participants’ adherence to the Mediterranean diet. Although there have been earlier attempts to compile MDS with absolute cut-offs [28, 29], no previous scoring systems have been constructed on such a sound evidence base as the one proposed by Sofi et al. [10]. The fact that one quarter of the pooled study sample scored more than ten points (about 60 % of the maximum score) suggests that the adherence to the Mediterranean diet in the these Eastern European cohorts was low, and among the three cohorts, dietary habits of Russians were the furthest from this pattern. This is not unexpected; however, estimation of this MDS in population samples from other countries or regions and comparison with our results is necessary to test the hypothesis that the adherence to Mediterranean diet in Eastern Europe is indeed lower than other populations.

Correlations between different versions of MDS have been reported to be weak to moderate [30]. In this light, the moderate agreement between the “traditional” MDS and the literature-based adherence score by Sofi et al., found in our study, was satisfactory, given the different cut-off values of component scores and also the arbitrary thresholds of the low, moderate and high scoring categories. The differences might be also partly due to the component which differed between the two scores (olive oil usage vs. unsaturated/saturated fatty acid ratio). Most effect estimates were stronger with the version using absolute cut-offs. The larger variation between individual MDSs, which is the result of the three-tier scoring system, is probably one of the primary reasons for this difference.

It has long been assumed that diet made important contribution to the high total and cardiovascular mortality in Eastern Europe, but it has been difficult to identify specific dietary factors responsible for this contribution. This study suggests that dietary patterns, as approximated by the MDS, are consistently associated with mortality outcomes and may well provide at least partial explanation for the contribution of nutrition to health status in the region.

## Electronic supplementary material

Below is the link to the electronic supplementary material.

Supplementary material 1 (PDF 279 kb)
