# Supplementary material for: Mediterranean diet score and total and cardiovascular mortality in Eastern Europe: the HAPIEE study
Source: Eur J Nutr. 2015 Nov 17;56(1):421–9. doi: 10.1007/s00394-015-1092-x (PMC5290049; doi:10.1007/s00394-015-1092-x)
Supplement: Supplementary file 1 — Supplementary material 1 (PDF 279 kb) [file 394_2015_1092_MOESM1_ESM.pdf]

SUPPLEMENTARY TABLES

Article Title: Mediterranean diet score and total and cardiovascular mortality in Eastern Europe: the HAPIEE study

Journal name: European Journal of Nutrition

Authors: Denes Stefler, Sofia Malyutina, Ruzena Kubinova, Andrzej Pajak, Anne Peasey, Hynek Pikhart, Eric J Brunner, Martin Bobak

Corresponding author: Denes Stefler; Department of Epidemiology and Public Health, University College London; denes.stefler.10@ucl.ac.uk

<u>Table S1 (Supplementary material).</u> Results of Cox-regression analysis between MDS and mortality outcomes by country cohort

Cause of death	Cohort	Death/n	MDS categories[a] Low	Moderate		High		PP (%)[b]	Per 1 SD[c] increase in MDS score		
			HR	HR	(95% CI)	HR	(95% CI)		HR	(95%CI)	p-value
Any-cause	Czech	364/5967	**1.0**	**0.72**	(0.56-0.93)	**0.82**	(0.62-1.09)	**7.1**	**0.94**	(0.85-1.05)	0.277
	Polish	388/6543	**1.0**	**0.83**	(0.64-1.06)	**0.78**	(0.59-1.05)	**8.4**	**0.90**	(0.81-0.99)	0.038
	Russian	562/6823	**1.0**	**0.94**	(0.79-1.12)	**0.98**	(0.75-1.29)	**0.7**	**0.97**	(0.88-1.05)	0.459
CVD	Czech	106/5965	**1.0**	**0.80**	(0.51-1.27)	**0.87**	(0.51-1.49)	**4.9**	**0.92**	(0.76-1.12)	0.424
	Polish	99/6517	**1.0**	**1.27**	(0.76-2.12)	**0.92**	(0.50-1.71)	**2.5**	**0.93**	(0.76-1.13)	0.458
	Russian	233/6781	**1.0**	**0.92**	(0.70-1.21)	**0.73**	(0.45-1.17)	**10.2**	**0.90**	(0.79-1.03)	0.117
CHD	Czech	43/5965	**1.0**	**1.28**	(0.60-2.73)	**1.10**	(0.44-2.72)	**-3.0**	**1.03**	(0.75-1.42)	0.841
	Polish	45/6517	**1.0**	**1.28**	(0.61-2.70)	**0.94**	(0.38-2.30)	**1.9**	**0.90**	(0.67-1.21)	0.481
	Russian	138/6781	**1.0**	**0.86**	(0.70-1.21)	**0.77**	(0.42-1.40)	**8.7**	**0.87**	(0.73-1.04)	0.120
Stroke	Czech	18/5965	**1.0**	**0.78**	(0.25-2.46)	**0.91**	(0.25-3.32)	**3.3**	**0.83**	(0.51-1.35)	0.450
	Polish	20/6517	**1.0**	**0.65**	(0.23-1.82)	**0.45**	(0.12-1.65)	**26.2**	**0.82**	(0.52-1.28)	0.378
	Russian	71/6781	**1.0**	**0.94**	(0.57-1.54)	**0.77**	(0.33-1.77)	**8.5**	**0.92**	(0.72-1.17)	0.490

All HRs are adjusted for age, sex, education, marital status, household amenities score, smoking, physical activity, total energy intake, vitamin supplement intake

[a] As in Table 2

[b] Preventable proportion of death if participants in the lowest two categories increased their adherence to Mediterranean diet one category upward

[c] 1SD=2.3 MDS points in the Czech, 2.2 MDS points in the Polish and 2.0 points in the Russian cohort

Table S2 (Supplementary material). Results of Cox-regression analysis between the "original" MDS[a] and mortality outcomes on the pooled sample

Cause of death	dead/n	model	MDS categories Low (0-3p)	Moderate (4-5p)		High (6-9p)		Per 1 SD[b] increase in MDS score		
			HR	HR	(95% CI)	HR	(95% CI)	HR	(95%CI)	p-value
Any-cause	1314/19,333	model1	**1.0**	**0.83**	(0.73-0.94)	**0.77**	(0.66-0.90)	**0.91**	(0.86-0.96)	0.001
		model2	**1.0**	**0.90**	(0.79-1.02)	**0.88**	(0.76-1.03)	**0.95**	(0.90-1.01)	0.108
CVD	438/19,263	model1	**1.0**	**0.79**	(0.64-0.98)	**0.69**	(0.53-0.90)	**0.86**	(0.79-0.95)	0.002
		model2	**1.0**	**0.86**	(0.69-1.07)	**0.81**	(0.62-1.06)	**0.92**	(0.83-1.01)	0.079
CHD	226/19,263	model1	**1.0**	**0.76**	(0.56-1.02)	**0.64**	(0.44-0.92)	**0.82**	(0.72-0.94)	0.004
		model2	**1.0**	**0.84**	(0.62-1.14)	**0.77**	(0.53-1.11)	**0.88**	(0.77-1.01)	0.065
Stroke	109/19,263	model1	**1.0**	**0.73**	(0.47-1.11)	**0.68**	(0.47-1.11)	**0.90**	(0.74-1.09)	0.269
		model2	**1.0**	**0.74**	(0.48-1.13)	**0.71**	(0.42-1.22)	**0.91**	(0.75-1.11)	0.369

model 1: adjusted for sex, age, cohort

model 2: adjusted for sex, age, cohort, smoking, education, household amenities score, marital status, energy intake, physical activity, vitamin supplement Intake

[a] Trichopoulou A, Orfanos P, Norat T et al (2005) Modified Mediterranean diet and survival: EPIC-elderly prospective cohort study. BMJ 330:991

[b] 1SD=1.5 points
